# Ecological and Health Effects of Lubricant Oils Emitted into the Environment

**DOI:** 10.3390/ijerph16163002

**Published:** 2019-08-20

**Authors:** Paulina Nowak, Karolina Kucharska, Marian Kamiński

**Affiliations:** Department of Process Engineering and Chemical Technology, Faculty of Chemistry, Gdansk University of Technology, 11/12 Gabriela Narutowicza Street, 80-233 Gdansk, Poland

**Keywords:** lubricating oils, mineral oils, chainsaw, harvester, forestry, open cutting system, biodegradability, OECD tests, environmental regulations

## Abstract

Lubricating oils used in machines with an open cutting system, such as a saw or harvester, are applied in forest areas, gardening, in the household, and in urban greenery. During the operation of the device with an open cutting system, the lubricating oil is emitted into the environment. Therefore, the use of an oil base and refining additives of petroleum origin in the content of lubricants is associated with a negative impact on health and the environment. The current legal regulations concerning lubricants applicable in the European Union (EU) assess the degree of biodegradability. Legislation permits the use of biodegradable oils at 60% for a period of 28 days. This means that, in practice, lubricating oil considered to be biodegradable can contain up to 50% of the so-called petroleum oil base. The paper aims to draw public attention to the need to reduce the toxicity and harmful effects, due to their composition, of lubricating oils emitted into the environment on health. The authors discuss the impact of petroleum oil lubricants on soils, groundwater, vegetation, and animals, and the impact of petroleum-origin oil mist on health. An overview of test methods for the biodegradability of lubricating oils is presented, including the Organization for Economic Cooperation and Development (OECD) 301 A–F, 310, and 302 A–D tests, as well as their standard equivalents. The current legal regulations regarding the use and control of lubricating oils emitted into the environment are discussed. Legal provisions are divided according to their area of application. Key issues regarding the biodegradability and toxicity of petroleum fractions in lubricating oils are also addressed. It is concluded that lubricating oils, emitted or potentially emitted into the environment, should contain only biodegradable ingredients in order to eliminate the negative impact on both the environment and health. Total biodegradability should be confirmed by widely applied tests. Therefore, a need to develop and implement low-cost and simple control procedures for each type of lubricating oil, ensuring the possibility of an indisputable conclusion about the presence and total absence of petroleum-derived components in oil, as well as the content of natural ingredients, occurs.

## 1. Introduction

Lubricating oil is considered as a fluid structural element of machines and devices. Its main task is to create a layer in the form of a microfilm between the moving elements of the device. Due to the specific properties, lubricating oil during operation can fulfill many functions, such as minimization of friction, elimination of scuffing of rubbing machine elements, washing of carbon deposits and micro particles, anti-corrosion, cooling, and other effects. Both environmental and application properties must be supported when the lubricants content is designed. Therefore, it must be characterized not only by an accurate biodegradability rate [1], but also, by appropriate physicochemical properties, such as an appropriate range of the viscosity index, dynamic viscosity at negative temperatures, melt temperatures, flash points, evaporability, as well as the basic or acidic number [2,3]. Lubricating oil is a mixture of base oil (>85%) and enriching additives [2,4,5,6].

The role of additives is to improve the quality of the lubricating oil. They are often products of an expensive organic synthesis process. As a rule, refining additives are synthetic, organic, or metal-organic chemicals, sometimes products of chemical processing of natural raw materials. The main types of additives are oxidation inhibitors, rheology modifiers, depressants, detergents and dispersants, de-emulsifiers and emulsifiers, lubricants, anti-wear, anti-seize agents, friction modifiers, corrosion and rust inhibitors, passivators, anti-foaming agents, dyes, fragrances, and multifunctional additives. The share of additives in the lubricating oil may range from a few ppm to several percent [4,5,6]. Currently, more and more enriching additives are created during advanced production technologies that favor the environment. Eco-friendly refining additives can become a beneficial alternative to petroleum-based products [7]. The base oil, which for the most part consists of modern lubricating oil, is a material with an oily consistency, characteristic chemical composition, and lubricating properties. Due to the origin, three main groups of oil bases (i.e., mineral oils, synthetic oils, and oils of natural origin) can be distinguished [2].

Most of the base oils used for the production of lubricating oils are produced during crude oil processing. This was due to the fact that these oils were characterized by a much lower cost than synthetic or natural base oils [8,9]. Oils produced from crude oil, commonly called mineral oils, are obtained in conventional oil refining processes. These are products of refining low-volatile oil fractions from vacuum distillation. In their composition they contain mainly mixtures of liquid low-volatile aliphatic hydrocarbons (branched) and alicyclic aliphatic substituted hydrocarbons, and a distillation temperature above 360 °C. Mineral oil contains many classes of chemical components, including paraffins, naphthenes, aromatic compounds, heteroatomic compounds, etc. [10]. Due to the different sources of petroleum origin and technologies of processing, mineral oils can have a diversified composition [11], that may include branched-chain aliphatic–aliphatic and alicyclic–alicyclic hydrocarbons, aliphatic or alicyclic aromatic hydrocarbons, mainly derivatives of benzene, but also biphenyl, diphenylmethane, triphenylmethane, naphthalene, anthracene, phenanthrene, and derivatives of chrysene [2]. The use of lubricating oil containing components obtained from crude oil has a negative impact on the environment and health [12]. It has become common to produce oil mixtures that improve the properties of mineral oils by adding natural oils [13].

Technological progress and the production trends of modern equipment and machines correspond with a constant increase in the improvements of energy efficiency and consumption. Due to the need to increase the efficiency of machines, there is an increase in demand for more efficient lubricants. Lubricating oils containing a synthetic oil base have been designed to maximize grease performance and bring measurable performance and economic benefits [10]. Synthetic bases such as poly-alpha-olefins (PAOs), alkylated aromatics, esters, polyglycols (PAGs), polybutenes, and polynectolefines (PIOs) are widely used in the lubricant industry [2,14].

Synthetic oils are produced in the processes of the synthesis of petrochemical industry raw materials or in the hydro catalytic process of hydrocarbon backbone conversion from natural gas [2]. In the world, over 80% of synthetic base oils are produced from three main classes of materials (i.e., poly-alpha-olefins (45%), esters, including dibasic esters, polyol esters (25%), and polyalkylene glycols (PAGs) (10%) [10]. The most popular fully-synthetic oils are PAO oils. This is due to the fact that oils of this type are characterized by a relatively high efficiency, but also relatively high price. Unfortunately, some synthetic oils can also pose a threat to the environment [8].

An increase in interest in the assessment of the impact of lubricating oils on the environment and health motivates research centers and industry to create new technologies for the production of fully biodegradable, lubricating oils of natural origin [2,15]. This applies especially to oils emitted into the environment, and those that may be there as a result of a failure. Lubricating oils with base oil and additives of vegetable origin (i.e., modern lubricating oils) appeared on the market in Europe in the mid 1980s. The acceptance and usage of these products, especially in the European forestry industry, is widespread and recently growing [9]. Thus, vegetable oils should have higher biodegradability ranging from 70–100% compared to other types of base oils [16]. Ingredients of vegetable base oils are triacylglycerols and their derivatives (i.e., diacylglycerols, monoacylglycerols, free fatty acids, and liquid (at room temperature) glycerol) [2]. These oils are natural products and; therefore, their chemical composition may differ when various crops are considered [9]. The main advantages of vegetable base oils include environmentally friendly values (e.g., fast and easy biodegradability and low toxicity to the aquatic environment) [17]. These oils are also characterized by good lubricity, high efficiency over a wide temperature range, high viscosity index and polarity, which ensures a high cleaning effect. More and more research concerns new bio-oil production technologies that can completely replace conventional lubricating oils [18,19]. Their low temperature properties and oxidative stability are the main disadvantages in comparison with mineral oil bases, and, unfortunately, additives are needed to overcome these problems. Moreover, vegetable oils differ in price, but in general they are about twice as expensive as petroleum oils [9].

Devices with an open cutting system, such as chainsaws and harvesters, are widely used in forest services, but also in households, in urban greenery, and during road works. Lubricating oils used in this type of equipment are emitted into the environment in the form of oil mist and micro droplets. The droplets cover and penetrate soils, ground waters, and may cause a strong effect on the ecosystem [20]. Therefore, the lubricating oil needs to be characterized by a fast and easy biodegradation. Due to the high requirements in the field of environmental protection, there is also a need to develop biodegradable base oils that are environmentally friendly and do not contain harmful ingredients [19]. The use of exclusively biodegradable oils should be included in legal regulations. That is why it is so important that adequate procedures of supervision and quality control of lubricating oils, especially those emitted into the environment, are applied in situ or during the production. It seems that the techniques that allow the analysis of the group composition of the final products are appropriate.

## 2. Impact of Oil Emissions

### 2.1. Environmental Issues

Lubricating oil is emitted into the environment in the form of oil mist and micro drops, posing a major threat to the environment. Strength and effects of interactions of oil derivatives are closely related to the composition, emission volume and frequency in a given area, and properties of a device with an open cutting system [21,22]. The biodegradability of mineral oils is very low. In the natural environment, oil of petroleum origin creates primary hazards for sawing operators, but also secondary hazards due to the accumulation of oils in plant, animal, and groundwater tissues [20]. Lubricating oils produced from crude oil are also a very significant threat to aquatic ecosystems. Water containing 1 ppm of oil is considered contaminated [23].

The film of petroleum substance on the water tank mirror may cause disturbances in the oxygen–gas exchange between the water and the atmosphere, but may also reduce the access of light to the depth of the tank. These limitations may lead to changes in the functioning and metabolic disturbances of aquatic organisms and, as a consequence, to the formation of an oxygen starvation area in the bottom parts of the reservoir, which may lead to ecosystem disorder. Reduction of the photosynthesis process, and increase of the water temperature by absorbing solar radiation, may threaten the proper development of aquatic plants and lead to the eutrophication of the reservoir [21]. Eutrophication of water reservoirs is a worldwide problem, occurring in rivers, lakes, and seas and even affecting oceans. Mineral oil contamination is a subject of public debate mainly when tanker disasters occur. Oil spills that spread in the water and the coast do not allow oxygen, which causes complete disappearance of life in a given area. The occurring changes are noticeable and; therefore, widely discussed. For example, the collision of two tankers (Atlantic Empress and Aegean Captain) caused an emission of ca 287,000 tons of fuel into the sea. The large oil spill flowed into the full Atlantic. Approximately 230,000 tons of oil got into the sea due to damage to the Amoco Cadiz oil tanker in 1978. However, one needs to remember that threats and problems of the modern world must also consider small emissions on wide areas. In this case; however, the biggest problem seems to be the lack of a real ability to estimate the amount of contamination and its effects.

Oil pollution also causes serious damage to soils, due to the multistep physicochemical processes leading to a change in the forms and distribution of organic matter, in the range of carbon, water, nitrogen, and phosphorus. As soil is an environment for a variety of microorganisms and higher living organisms, its contamination with petroleum-based lubricants becomes hazardous and a detrimental effect on biological life may occur. The proper functioning of the ecosystem may be disturbed. Mineral oil can clog pores in the soil, resulting in reduced aeration and water infiltration. The presence of petroleum compounds may reduce or limit the permeability of soils, and, consequently, cause the degradation of soils due to oxygen deficit [24,25].

### 2.2. Health Issues

Eco-toxicity, incomplete biodegradability, and highly probable carcinogenicity of base oils derived from crude oil is an object of public debate regarding long-term health effects of oil application and emission [26]. The routes of penetration of toxic substances from oil mist into the human body are mainly respiratory and skin. Oil mist generated during the operation of the device with the open cutting system gets through the respiratory tract, causing changes in the lungs, but also in the liver, kidneys, adrenal glands, and heart [20]. It is also absorbed by the skin, causing significant health consequences. These include irritant and allergic reactions. People exposed to long-term contact with oil mist emitted during operation of the cutting device show higher incidence of cancer, including, most often, skin cancer [20,26].

The saw chain operator, through the nature of his work, especially in long-term work, such as logging, stays in an environment with a high level of contamination of toxic substances. The oil mist may contain aromatic hydrocarbons, polycyclic aromatic hydrocarbons (PAHs), but also benzene, toluene, or methylbenzene, which a negative influence on the respiratory and nervous systems of the device operator. As shown by research, typical complaints reported after the work of the device operators, are irritations of the eyes and upper respiratory tract, headaches, and fatigue [27,28]. Petroleum oils are known carcinogens, and medical records indicate that they cause uncomfortable eczema and oil acne. In addition, prolonged exposure to oil-based oil mist may cause irritation to the respiratory tract [9].

In addition, most of the petroleum components are slowly degraded and at the same time subjected to unavoidable interactions with environmental components. Derived products are transformed due to chemical reactions under the influence of sunlight, with reactions with oxygen from the air, as well as with water and soil components. The newly-formed secondary chemicals are mostly much more eco-toxic and often more harmful to health than the original chemical forms. It is estimated that derivatives formed as a result of bioconversion of substances emitted into the environment may also be more harmful than their original precursors [20,29]. The lack of an effective way to dispose the exhausted lubricating oil also occurs as a problem [30].

### 2.3. Environmental Cost

Estimation of environmental costs is a necessary step for a full economic analysis of the use of wood resources, because it is associated with the introduction of pollutants into the environment, as well as with the use of energy. Environmental costs are a general concept for various costs related to environmental protection and environmental impact. In practice, they are recognized in the calculation of the operating costs of enterprises in a piecemeal manner or are included in aggregate items. So far, harmonized standards have not been developed in terms of measurement principles, documentation, records, and settlement of environmental expenditure in the enterprise. Reporting obligations of entrepreneurs, covering environmental protection, are regulated by numerous legal acts. With regard to environmental costs, there is no comprehensive research specifying their amount and structure in the entire national economy. The Central Statistical Office (Poland) conducts only periodic and fragmentary surveys in this respect, based on data provided by a small number of enterprises. The process of environmental protection includes the use of environmental resources, prevention of threats (preventive activities), limiting the emission of threats (reduction activity), repairing damage (restitution), and managing this activity. Actions for environmental protection require consumption of material and financial resources, labor resources, and use of external services, as a result of which appropriate material and financial results are generated. Resource consumption can be quantitatively measured (e.g., using cost-ratings) [31]. With regard to environmental protection, the notion of costs applies, due to the occurrence of pure cash expenditures. In practice, the differences between costs and expenditures incurred for environmental protection are often discussed. Depending on the decision-making aspects, it may be significant expenditures on investments to protect the environment; other times, depreciation costs of non-current assets used to protect the environment.

Currently, wood and wood-based materials have about 30,000 applications. They are used in construction, mining, and power engineering, for the production of agricultural and industrial machines, musical instruments, floors, furniture, plates, paper, tools, packaging, sports equipment, toys, and office supplies. The size of the harvested timber is determined by the forest management plan, which is prepared for each forest inspectorate for a period of 10 years. This allows the timber to be harvested within the limits of the production capacity of a given forest.

Harvested wood is the basic source of all wood and its acquisition and processing has a noticeable impact on the environment. Wood market leaders are located first of all in Asia, followed by Africa and Europe. Global powers in obtaining wood due to the largest forest area (in percentage of the total area of forests in the world) are Russia, Brazil, Canada, USA, and China. As Europe is considered, Sweden, Germany, France, and Finland have the highest potential to acquire timber. In Europe, the basic legal requirements for forestry operations are compliance with Forest Stewardship Council (FSC) and Program for the Endorsement of Forest Certification (PEFC) systems [32].

The annual harvesting of wood in the world totals approximately 3,713,681 thousand m^3^ [32]. A chainsaw needs 0.05 L of lubricating oil to obtain 1 m^3^ of wood, while a harvester needs less than half the lubricating oil (i.e., only 0.02 L). Assuming that, on average, 1 m^3^ of secondary oil needs 0.03 L of lubricating oil, this would mean that 117,576,000 L of oil are emitted into the environment annually (Table 1).

The majority of lubricating oils, due to the cost of production in their composition, have a base oil of petroleum origin. Assuming that 60% of oil-based lubricants are used annually, 70 million liters of harmful lubricating oil are emitted when the world scale is considered. One liter of this type of oil makes it unfit to consume a million liters of drinking water (i.e., 600 million liters of water may be contaminated during tree harvesting per year) [23]. The cost of the soil and groundwater remediation can be; therefore, considered enormous. Remediation of soils from petroleum contaminants is a difficult and long-lasting process [33]. The quality of soils and the degree of oilification corresponds with the quality of groundwater. For penetrating soils, the consequences of oiling can be irreversible. If solid vegetation occurs in the contaminated area, it may help to regulate the flow of water to surface waters. Water is absorbed from the soil by the roots of plants and released into the atmosphere from the leaves—a process known as evapotranspiration. Plant roots also bind the soil and protect it from erosion. Rainwater and melted snow flow freely from the harvest, causing a higher flow of the baseline and increasing the likelihood of flooding. Unprotected soil is easily washed away, and disruption of the soil by other activities such as road construction may exacerbate this problem. These results can have a devastating effect on aquatic organisms that have adapted the lifestyle to natural flow and sludge conditions. For example, high spawning flows can wash off fish eggs deposited on the bottom of the stream. Many aquatic invertebrates require gravel or sand to live and will not tolerate the muddy bottoms of the stream. High flow and poor water clarity can also affect the ability of fish and invertebrates to capture victims. Problems caused by erosion are not limited to freshwater habitats, because sediments travel downstream and accumulate in estuaries.

Table 2 summarizes the main options used for separating and removing oils, including lubricating oils from various types of environments. The unit processes and operations listed in the table should become one of the exponents of the environmental cost that the harvesting company must incur during the acquisition and processing of wood.

## 3. Current Legal Regulations

Oil spills pollute the soil and water. Governments, in cooperation with industry, develop standards, regulations, and procedures to reduce the risk of accidents and leaks. However, in the case of streams introduced into the environment in the form of oil mist, such limitations are physically impossible, hence the need to introduce legal regulations regarding the composition of the indicated oils. Legal regulations, in the case of emissions to the environment, should allow application of eco-friendly, completely biodegradable, and harmless (to the environment and health) lubricants, preferably of natural origin (based on vegetable oils). Lubricating oils should not; however, contain even small amounts of petroleum components, because they have a negative impact on the environment and health. Some countries are aware of the existing problem. In Germany and Scandinavia, there are about 80 brands of lubricants produced on the basis of vegetable oils. In Austria, all mineral oil-based chainsaw oils are prohibited [9].

### 3.1. Regulations in Poland

In Poland, the Decision of the General Director of Polish National Forests no. 148 (October 2018) regarding access to uniform document templates for forestry services ordering in forest management at the State Forests organizational units, determines that “the contractor is obliged to equip all machines, tractors, and equipment working on forest surfaces with appropriate sets (sorbents, sorption mats, etc.) for absorbing spilled fuel or oil and other process fluids used in machines, tractors, chainsaws, and other devices working in the forest and using these resources in situations requiring application (failures, repairs refueling etc.) to prevent environmental contamination. It is allowed to have and use of canisters with safe tips (dispensers), preventing spilling (overflow) of oil and fuel mixture during refueling of the saw (substitute for fuel and oil absorbing mats)” [37]. In 2017, more than one million lubricating oils were emitted into the forests when logging timber through the Polish State Forests [32]. However, the amount of petroleum components that were part of the oils used is unknown.

### 3.2. Regulations in European Union

The Coordinating European Council (CEC) has established a methodology for biodegradation tests. Current methods of testing lubricating oils, emitted into the environments, include recommendations for their biodegradability using standard tests. The European Commission Regulation (EC) No. 440/2008 of 30 May 2008, which contains methodologies for biodegradability assessment with the use of tests developed by the Organization for Economic Cooperation and Development (OECD 301 A–F), is in force in the European Union, establishing methods in accordance with Regulation (EC) No 1907/2006 of the European Parliament and of the Council on the Registration, Evaluation, Authorization, and Restriction of Chemicals (REACH) [1,38]. OECD 301 A–F methods assess biodegradability by subjecting the test oil to specific microorganisms, most often for 28 days. Tests allowing a degradation value of 60% at a given time (28 days) may be inadequate for lubricating oils, because the remaining 40% of substances can be subjected to natural biodegradation over even several hundred years, and this does not solve the issue of emissions, even though its scale is reduced [20,29,39].

#### Revision of European Union Ecolabel Criteria for Lubricants

The regulations enforced in Poland, as well as in many other countries of the European Union, apart from Scandinavia, allow some of the oils on the market to contain up to approximately 50% of the mineral oil base and still be considered as biodegradable, according to the Decision of the European Commission of 24 June 2011, regarding the award of the ecological label in the European Union to oils and lubricants. Still, commercially available lubricating oils containing up to 50% of petroleum-based components are referred to as biodegradable, which can be misleading for the user deciding to buy them. Thus, an unaware exposure occurs regarding health and the environment [17]. It should be added that there are also producers of lubricating oils in Poland that do not contain components made of crude oil (i.e., oils that are completely “friendly” to the environment). The problem is that this oil is more expensive, especially if it contains completely biodegradable additives and bio-components with a high production cost.

## 4. Biodegradability

Lubricants that enter the environment or which are subjected to emissions to the environment, must meet the biodegradability criteria developed by OECD in 301 A–F tests, which determine the potential biodegradability and properties of oil [40]. Biodegradation is an ambiguous concept, no objective definition has been developed; therefore, it should be determined which test is appropriate for the test substance and adopt the assessment criteria. During the biodegradation process the molecules of substances are decomposed, as a result of the complex interaction of living organisms as well as their enzymes in a specific environment (oxygen, anaerobic, in soil, in water) [17]. OECD biodegradability tests utilize standard soil bacterial strains and specific standard test conditions. To assess the biodegradability of lubricating oils, a test is used to assess the rapid or potential biodegradability of the substance. Table 3 presents the tests used to assess biodegradability.

The assessment of the biodegradability of lubricants is not straightforward, because they are practically insoluble in water. In addition, the composition of the lubricating oil is varied, depending on the components and the set of refining additives; therefore, it is also necessary to select the appropriate test for the assessment of biodegradability and the use of an appropriate method of sample preparation [41]. Due to the low biodegradability of mineral oils, corrective action is required if leaks occur. Various pollutants can be degraded by microorganisms, unfortunately these types of biological processes are expensive, especially when compared to conventional pumping and purification processes used for the reclamation of contaminated groundwater. Experimentally, in 1997–2000, biological remediation techniques were applied and an impressive biodegradation factor of 86% was noted over a thousand days on an area of 500 m^2^, up to a depth of 10 m contaminated with cable insulation oil [49].

A similar study to show the potential of bioremediation of contaminated soils was carried out in Boucherville, Quebec, where a leakage of transformer oil was recorded [50]. According to reported successes, studies to optimize the isolation of microbial strains that have enzyme systems that degrade and use oils as carbon and hydrogen sources may be a possible direction of development [51]. Strains belonging to the genus *Bacillus* have an impressive biodegradability, using crude oil as the sole source of carbon and energy even in harsh environmental conditions [30]. Certain parameters, such as the oil brand, the oil concentration, its degree of emulsification, and aeration, affect the microbiological distribution of the oil [52]. Surfactants belonging to acylated fatty acids or amino acids accelerate the microbial degradation of mineral oils. More complete degradation of mineral oils is identified as being caused only by interfacial activity [53].

According to the methods included in the legal regulations, it should be checked whether the substance is prone to rapid biodegradation. OECD 301 A–F tests as well as OECD 310 tests are used to assess rapid biodegradation. Tests are performed over a period of 28 days, using stricter conditions, including low concentrations of inadvertent inoculum pre-conditioned to the test substance. When a substance undergoes a high level of biodegradability, it should be considered that it will degrade in the environment in a short time. However, if the substance is not prone to rapid biodegradation, it should be subjected to further testing. OECD 302 A–C tests are used to assess potential biodegradability, which aims to assess the potential biochemical distribution expressed in percent and thus predict the rate of disappearance of the test substance in the environment. For more information on the degradation rate of the substance in the environment, OECD 303 A and B tests are recommended. The 301 and 310 series tests allow you to track the rate of biodegradation and determine if the substance is easily biodegradable. The substance is considered to be biodegradable if it reaches the required biodegradation level of 70% within 28 days (in the case of determining the loss of dissolved organic carbon (DOC/TOC). The substance may also be considered as biodegradable if it reaches the level of biodegradation to at least 60% (in the case of biological oxygen demand (BOD5) or produced CO_2_). One needs to consider, that the day on which 10% of the substance volume is biodegraded should not be longer than 10 days [17,39,41].

Oil meeting the requirements of the OECD 301 B test should be biodegradable under test conditions above 60% for 28 days. It is assumed then that the total distribution of the remaining part of the analyte will take place. Research results indicate that this assumption is valid only for synthetic and natural oils. On the other hand, most of the components derived from crude oil are slowly degraded and, at the same time, undergoes various chemical reactions [29]. Therefore, it seems necessary to have a multi-stage control covering a period of time significantly longer than 28 days. In addition, the methods of biodegradability testing are time-consuming and expensive. In practice, it is very difficult to use them in the “daily” technical inspection of oils, where often the decision to allow the oil for use needs to be issued on a regular basis.

## 5. Biotoxicity

In relation to the physicochemical properties of mineral oils, these substances are strongly hydrophobic and slightly reactive. As organic substances that do not enter the metabolic pathways, oils and solvents produced on the basis of mineral oils are excreted intact with feces and to some extent in the urine. However, there are reports of a small absorption of this type of compound in the small intestine [54]. In 1966, Ebert et al. showed, in an experiment using radio-labeled mineral oil and rats, that this absorption is 1.5% of the obtained dose [55]. In the body, after absorption, mineral oils are distributed, with the participation of connective tissue, to such organs as the liver, spleen, mesenteric lymph nodes, and subcutaneous tissue [56]. The secondary metabolism of these substances in hepatocytes may lead to their degradation into derivatives that affect the overall mechanism of lipids in the body [57]. The risk associated with inhalation exposure has been studied in animal populations [58]. The studies show that, unlike subcutaneous and fatty tissue, the connective tissue and brain tissues are removed relatively quickly.

The complexity of the mineral oil-based hydrocarbon structure precludes identification of toxicological hazards based on the properties of a single substance. Toxic kinetics in living organisms, is concluded on the basis of literature reports [59]. The oral absorption of mineral oils decreases with increasing number of carbon atoms. Hydrocarbons with a chain length over 50 carbons do not show absorption in living organisms. Branched alkanes are slightly less absorbed than linear and cycloalkanes of comparable molecular weight [60]. Alkanes are metabolized to fatty alcohols and potentially to fatty acids. Linear alkanes are metabolized more easily than cyclic and branched alkanes. Hydrocarbons with a number of carbon atoms between C16 and C45 can accumulate in the body. High accumulation potential was observed in Fischer 344 rats. In humans, accumulation in various tissues such as adipose tissue, lymph nodes, liver, and spleen is observed [61]. The current ADI (acceptable daily intake) values presented by WHO in 2002 are 0–0.01 mg/kg body weight per day for medium and low viscosity oils, which are mainly based on the results obtained from experiments with Fischer 344 rats. No ADI values have been proposed for inhalation oils to which professional groups particularly are exposed.

The debate on the toxicity of mineral substances accumulating in living organisms mainly concern those substances that are taken in food. It is also known that paraffins contained in cosmetics and ointments are transferred to human milk through the skin of the breast. The mother passes about 1% of the mineral paraffins accumulated in her body to the child [60]. It should; therefore, be assumed that mineral oils can also be absorbed through the skin from oil mist sprayed during the operation of saws. The influence of these types of mineral oils inhaled from the surrounding air remains unknown.

## 6. Concluding Remarks

It is worth paying attention to the problem of emission of lubricating oils produced from crude oil to the environment. Some literature sources state that every year, during forestry work using chainsaws, as much as 7 million liters of various types of mineral oils enter the soil [35]. Since the oils used for spreading have a significant impact on the environment and health, they should only contain completely biodegradable ingredients. Oils produced from crude oil have a negative impact on the environment, cause serious contamination of soils, groundwater, and can accumulate in plant tissues as well as terrestrial and aquatic animals. In addition, they are a significant health risk, causing numerous allergic reactions and diseases of the nervous and respiratory systems, and long-term contact with oil mist may be the cause of cancer. Environmental lubricating oils used in open cutting systems, such as chainsaws or harvesters, should contain only biodegradable components. Ingredients of lubricants produced from crude oil have a negative impact on the environment and health; therefore, their use in open cutting systems should be prohibited and punished. Methods of biodegradability tests for oils should confirm total biodegradability. Therefore, there is a need to develop and implement, as technical control methodologies, low-cost and uncomplicated control procedures for each type of lubricating oil, providing the possibility of indisputable inference about the presence and content, or the total absence of oil-derived components in oil, as well as the content of natural ingredients.

The legal regulations regarding lubricants need to specify the speed and biodegradability potential of lubricants. Unfortunately, current methodologies do not report 100% biodegradability of the substance and the methods of biodegradability assessment are time-consuming and expensive. Therefore, there is a need to develop and implement low-cost and simple control procedures for each type of lubricating oil, ensuring the possibility of an indisputable conclusion about the presence and total absence of petroleum-derived components in oil, as well as the content of natural ingredients.

Moreover, legal regulations should require the use of only fully and easily biodegradable lubricating oils, especially those emitted into the environment. Bio-lubricants have emerged as a potential and viable way to replace, totally or partially, mineral oils due to their effectiveness in the boundary lubrication regime for different applications, including devices that work with an open system, such as a cutting saw or harvester [62].

## Figures and Tables

**Table 1:** Comparison of harvesting of wood and used oil divided into continents and specified for Poland in 2017 [32].

Continental	Harvested Wood	The Amount of Lubricating Oil Used
	(Thousand m^3^)	(Thousand L)
Asia	1,117,409	33,522
Africa	737,603	22,128
Europe	725,645	21,769
Poland	42,200	1266
Russia	205,507	6165
North America	568,915	17,067
Central and South America	489,982	14,699
Oceania	74,128	2224

**Table 2:** Main options available for separation and disposal of oil and debris [30,34,35,36].

Type of Contaminated Material	Separation Method	Method of Development
Oil mixed with wood, plastics, seaweed	Collection of liquid oil during temporary storage; Oil flushing with water; Removal of free water compression	Stabilization and reuse after removal of plastics and large pollutants; Degradation through tillage or composting in the case of oil mixed with seaweed or crustaceans; Landfill combustion
Oil mixed with pavement, pebbles, or shingle	Collection of liquid oil from material during temporary storage Extraction of oil from material	Restoration of cleaned stones to the source; Stabilization and reuse of oil; Dumping; Use of recovered liquid oil as fuel or refinery raw material; Return of treated water to the source Stabilization and reuse; Degradation through soil cultivation or composting; Storage of waste; Combustion
Non-emulsified oils and sewage	Sedimentation and gravity separation of free water The recovered water may require further processing or filtration	Use of recovered oil as fuel or refinery raw material; Return of treated water to the source

**Table 3:** Tests used to assess the rapid and potential aerobic biodegradability of various materials/chemical compounds in water [17,39,41].

OECD Test	International Organization for Standardization (ISO) Norm	Parameter Monitored	Ref.
Rapid biodegradability assessment tests			
301 A DOC Die-Away	Water quality. Evaluation in an aqueous medium of the “ultimate” aerobic biodegradability of organic compounds. Method by analysis of dissolved organic carbon (DOC).	DOC ^1^	[42]
301 B CO_2_ Evolution Test	Water quality. Evaluation of ultimate aerobic biodegradability of organic compounds in aqueous medium. Carbon dioxide evolution test.	CO_2_ Production	[43]
301 C MITI I Test Modified MITI I test	No equivalent. Test used in Japan.	BOD5 ^2^	-
301 D Closed bottle test	Water quality. Evaluation in an aqueous medium of the “ultimate” aerobic biodegradability of organic compounds. Method by analysis of biochemical oxygen demand (closed bottle test).	BOD5 ^2^	[44]
301 E Modified OECD ^3^ Screening Test	Water quality. Evaluation of the “ready”, “ultimate” aerobic biodegradability of organic compounds in an aqueous medium. Method by analysis of dissolved organic carbon (DOC).	DOC ^1^	[42]
301 F Manometric Respiratory Test	Water quality. Evaluation of ultimate aerobic biodegradability of organic compounds in aqueous medium by determination of oxygen demand in a closed respirometer.	BOD5 ^2^	[45]
310 CO_2_ Headspace Test	Water quality. Evaluation of ultimate aerobic biodegradability of organic compounds in aqueous medium. Method by analysis of inorganic carbon in sealed vessels (CO_2_ headspace test).	CO_2_ Production	[46]
Tests for potential biodegradability.			
302 A Modified SCAS test	Water quality. Evaluation of the aerobic biodegradability of organic compounds in an aqueous medium. Semi-continuous activated sludge method (SCAS).	DOC ^1^	[47]
302 B Zahn-Wellens modified test	Water quality. Evaluation of ultimate aerobic biodegradability of organic compounds in aqueous medium. Static test (Zahn-Wellens method).	DOC ^1^	[48]
302 C Modified MITI ^4^ II test	No equivalent. Test used in Japan.	BOD5 ^2^	-
302 D CONCAWE ^5^ Test	No equivalent.	CO_2_ Production	-

^1^ DOC—(dissolved organic carbon) is the fraction of total organic carbon operationally defined as that which can pass through a filter size that typically ranges in size from 0.22 and 0.7 micrometers; ^2^ BOD—(biochemical oxygen demand) is the amount of dissolved oxygen needed by aerobic biological organisms to break down organic material present in a given water sample at certain temperature over a specific time period; ^3^ OECD—Organisation for Economic Co-operation and Development; ^4^ MITI—Ministry of International Trade and Industry; ^5^ CONCAWE—the European oil companies’ organization for environment, health and safety.
